# Engineered Cleistogamy in *Camelina sativa* for bioconfinement

**DOI:** 10.1093/hr/uhac280

**Published:** 2022-12-22

**Authors:** Debao Huang, Liwei Gao, Jeremy McAdams, Fangzhou Zhao, Hongyan Lu, Yonghui Wu, Jeremy Martin, Sherif M Sherif, Jayasankar Subramanian, Hui Duan, Wusheng Liu

**Affiliations:** Department of Horticultural Science, North Carolina State University, Raleigh, NC 27607, USA; Department of Horticultural Science, North Carolina State University, Raleigh, NC 27607, USA; College of Life Sciences, Ganzhou Normal University, Ganzhou, Jiangxi 341000, China; Department of Horticultural Science, North Carolina State University, Raleigh, NC 27607, USA; Department of Horticultural Science, North Carolina State University, Raleigh, NC 27607, USA; National Center for Soybean Improvement, State Key Laboratory of Crop Genetics and Germplasm Enhancement, Nanjing Agricultural University, Nanjing, Jiangsu 210095, China; Department of Horticultural Science, North Carolina State University, Raleigh, NC 27607, USA; College of Food Science and Engineering, Wuhan Polytechnic University, Wuhan, Hubei 430048, China; Department of Horticultural Science, North Carolina State University, Raleigh, NC 27607, USA; Sandhills Research Station, North Carolina State University, Jackson Springs, NC 27281, USA; Vineland Research Station, Department of Plant Agriculture, University of Guelph, Vinland Station, ON LOR 2E0, Canada; Alson H. Smith Jr. Agricultural Research and Extension Center, School of Plant and Environmental Sciences, Virginia Tech, Winchester, VA 22602, USA; Vineland Research Station, Department of Plant Agriculture, University of Guelph, Vinland Station, ON LOR 2E0, Canada; Alson H. Smith Jr. Agricultural Research and Extension Center, School of Plant and Environmental Sciences, Virginia Tech, Winchester, VA 22602, USA; Department of Horticultural Science, North Carolina State University, Raleigh, NC 27607, USA; USDA-ARS, U.S. National Arboretum, Floral and Nursery Plants Research Unit, Beltsville Agricultural Research Center (BARC)-West, Beltsville, MD 20705, USA

## Abstract

*Camelina sativa* is a self-pollinating and facultative outcrossing oilseed crop. Genetic engineering has been used to improve camelina yield potential for altered fatty acid composition, modified protein profiles, improved seed and oil yield, and enhanced drought resistance. The deployment of transgenic camelina in the field posits high risks related to the introgression of transgenes into non-transgenic camelina and wild relatives. Thus, effective bioconfinement strategies need to be developed to prevent pollen-mediated gene flow (PMGF) from transgenic camelina. In the present study, we overexpressed the cleistogamy (i.e. floral petal non-openness)-inducing *PpJAZ1* gene from peach in transgenic camelina. Transgenic camelina overexpressing *PpJAZ1* showed three levels of cleistogamy, affected pollen germination rates after anthesis but not during anthesis, and caused a minor silicle abortion only on the main branches. We also conducted field trials to examine the effects of the overexpressed *PpJAZ1* on PMGF in the field, and found that the overexpressed *PpJAZ1* dramatically inhibited PMGF from transgenic camelina to non-transgenic camelina under the field conditions. Thus, the engineered cleistogamy using the overexpressed *PpJAZ1* is a highly effective bioconfinement strategy to limit PMGF from transgenic camelina, and could be used for bioconfinement in other dicot species.

## Introduction

*Camelina sativa* (L.) Crantz. (camelina or false flax; allohexaploid; 2*n* = 6*x* = 40) is native to Europe and Central Asia, and cultivated as a re-emergent oilseed crop in Europe, Asia, and North America [1]. It has a great potential for the production of biodiesel, jet fuel, beneficial omega-3 lipids, bioplastics, and animal feed due to its high (36–47%) seed oil contents with high (> 90%) unsaturated fatty acids [1–4]. It possesses valuable agronomic traits such as a short (85–100 days) life cycle, low input requirements, adaptability to adverse environments, and resistance to the primary disease-causing fungi [1, 5, 6], flea beetles [7, 8], and insects [9] infesting Brassicaceae. These excellent agronomic properties permit camelina to grow as a spring or winter annual on marginal lands or as part of a multiple crop rotation system.

Wild camelina is present in 28 U.S. states and commercial production of camelina varieties in the U.S. centers on Montana, Colorado, Oregon, Washington, and Wyoming with the majority of the production being sold to the U.S. Air Force under contract [6]. Varietal testing and evaluation has been conducted in California, Kentucky, Iowa, Florida, North Carolina, Arizona, Colorado, Washington, Nevada, Montana and Wyoming [6, 10–12]. Meanwhile, genetic engineering has been used to improve camelina’s yield potential of altered fatty acid composition [13–31], modified protein profiles [32], increased carotenoid content [33], improved seed and oil yield [25, 34–36], and enhanced drought resistance [35, 37] and other osmotic stress tolerance [38]. More importantly, field testing of genetically engineered (or transgenic) camelina has been conducted in Canada [39, 40], U.K. [41], and Michigan [42, 43].

**Figure 1:**
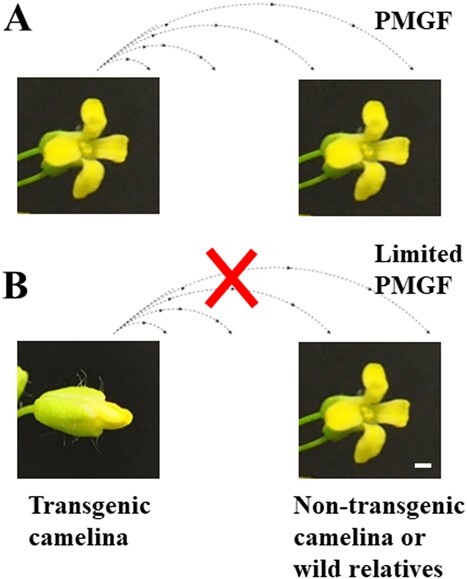
Illustration of the effects of the fully opened (A) or cleistogamous (non-opening) (B) flowers on PMGF from transgenic camelina to non-transgenic camelina or wild relatives. Red cross, inhibited PMGF. Bar = 1 mm.

The inclusion of transgenic camelina into the agricultural landscape carries high risks related to the introgression of transgenes – especially drought resistance genes and selectable marker genes – into related agricultural and wild relatives. Camelina is a predominantly self-pollinating species and a facultative outcrossing species [6]. A small-scale field trial conducted by Walsh *et al.* [39] revealed that pollen-mediated gene flow (PMGF) from transgenic camelina to non-transgenic camelina was 0.28% at close proximity (up to 0.6 m) when the pollen donor area was small (0.2 × 7.0 m). A medium-scale field test detected a maximum PMGF of 0.78% at the minimum sampling distance (0.2 m), which produced 7.8 hybrid seeds per plant [40]. This is apparently higher than the PMGF in soybean, which is 0.52% average frequency in the non-transgenic soybean plants located at one meter from the transgenic plants [44]. It is expected that the intraspecific PMGF in camelina will be much higher at a commercial scale than observed in the small- or medium-scale field studies [39, 40], partially due to the presence of massive pollen recipients. In addition, up to 29 different insect species were observed visiting camelina in field trials in Germany [45], raising a concern about insect-mediated intraspecific and interspecific PMGF in camelina. Moreover, hybridization experiments revealed a high, moderate, and low level of interfertility (i.e. the resulting hybrids are fertile) with camelina’s wild relatives *C. alyssum*, *C. microcarpa*, and *C. rumelica*, respectively [46]. Considering these *Camelina* species, including camelina itself, are widely naturalized weeds in the U.S., effective bioconfinement technologies need to be developed to prevent PMGF from transgenic camelina to non-transgenic camelina and the wild relatives (Figure 1). Transgene flow and regulatory issues make bioconfinement necessary for sustainable deployment of transgenic camelina in the field.

Potential bioconfinement strategies for restricting PMGF include plastid transformation, male sterility, delayed and decreased flowering, post-zygotic barriers to transgene introgression, transgene excision and mitigation, creation of selectively terminable transgenic lines, genetic use restriction technologies, cleistogamy, etc. [47–56]. Among these methods, the inclusion of cleistogamy is unique in making flowers self-pollinating without petal opening during pollen shedding (Figure 1B). It can maximally restrain pollen spread out of the flowers, and transgenes can be well-restrained within the closed petals. This trait is highly attractive for the efforts to maintain genetic purity and to generate transgenic crops with a low risk of PMGF if the trait does not interfere with other agronomic traits [49, 57]. Several cleistogamous mutants have been identified, including the *cl7(t)* [58], *d7* [59], *ld(t)* [60], and *superwoman1-cleistogamy* (*spw1-cls*) [61] mutants in rice, the *cleistogamy 1* (*Cly1*) mutant in barley [57, 62, 63], and the *Bn-CLG1A-1D* [64], *Zhong9*-*Clg* [65], and *BnaC03.FBA* [66] mutants in canola (*Brassica napus*). The rice *cl7(t)* mutant was obtained through ethyl methanesulfonate (EMS) mutagenesis and has normal floral organs [58]. The rice *d7* [59] and *ld(t)* [60] mutants originated from spontaneous mutations and have abnormal glumes and missing lodicules, respectively. The mutated genes in these three mutants have not been identified yet. The rice *superwoman1-cleistogamy* (*spw1-cls*) mutant was generated by the random mutagenesis with N-methyl-N-nitrosourea (MNU) and lacks lodicules or shows lodicule deformity [61]. A single base change leading to an isoleucine to threonine substitution (I45T) in the MADS-box domain of the *SPW1* gene reduces the gene’s interaction ability with its dimerization partners MADS2 and MADS4, resulting in the cleistogamous mutant phenotype. In barley, a spontaneous synonymous nucleotide substitution in the barley *AP2* gene mutated the miR172 target site, leading to the cleistogamous *Cly1* phenotype due to the failure of the lodicules to develop properly [57, 62, 63]. In canola, an EMS-induced C-to-T nucleotide transition converted the amino acid proline (P) at position 325 to a leucine (L) (i.e. P325L) in the *Bn-CLG1A* gene (A stands for the A subgenome), which encodes a RINGv E3 ubiquitin ligase [64]. The mutation caused a cleistogamous phenotype under the control of its native promoter due to a significant negative regulation of cutin biosynthesis/loading and thus an inhibition of petal development [64, 67, 68]. In addition, *Zhong9*-*Clg* is another EMS-induced cleistogamous mutant in canola (the mutated gene has not been identified yet) [65], while *BnaC03.FBA* in canola contains a miniature inverted-repeat transposable elements (MITEs)-mediated chromosome inversion, resulting in a high tissue-specific expression of the *BnaC03.FBA* gene [66].

To date, only the rice *spw1-cls* mutant [69] and the canola *Bn-CLG1A-1D* mutant [64], which were generated via random mutagenesis, have been used in field trials to examine their effects on inhibiting PMGF between pollen donors and recipient lines. The cleistogamy of the rice *spw1-cls* mutant is an effective tool for bioconfinement of transgenes in rice without interference with agronomic performance (such as yield) under the field conditions [69]. However, Leflon *et al.* [64] found that the canola cleistogamy phenotype was not stable in the field. Thus, cleistogamy-inducing genes are not available for bioconfinement of transgenes in dicotyledonous plants yet.

In our previous report [70], we identified a *JAZ1* gene, a repressor gene in the jasmonic acid (JA) signaling pathway, regulates petal openness in the non-showy peach (*Pronus persica* (L.) Batsch) during anthesis. We found that ectopic expression of *PpJAZ1* converted the opening tobacco flowers into cleistogamous flowers without interference with seed yield [70]. It was reported that JA is involved in petal expansion and a low level of JA was detected in Arabidopsis mutants with unopening petals and in Chinese cabbage with degenerated petals [71–73]. JAZ1 contains the conserved ZIM and Jas domains, which interact with different partner proteins in JA signal transduction [74]. In Arabidopsis, overexpression of a truncated *JAZ1* gene lacking the Jas domain showed irresponsiveness to the JA inhibition of root growth and a failure in pollen germination [75].

In the present study, we overexpressed the peach cleistogamy-inducing *PpJAZ1* gene in camelina, and examined the overexpression phenotype in stable transgenic camelina lines under greenhouse conditions. We also examined the PMGF from transgenic camelina to non-transgenic camelina under the field conditions, which showed that the engineered cleistogamy dramatically restricted PMGF from transgenic to non-transgenic camelina in the field. As a result, clestogamy engineering using the *PpJAZ1* overexpression is a highly effective bioconfinement strategy to restrict PMGF for sustainable deployment of transgenic camelina for seed production in the field. This study is the first to examine the effects of the overexpressed *PpJAZ1* gene in a dicot plant species other than tobacco and use it for bioconfinement of transgenic pollen under the field conditions.

## Results

### Generation of stable transgenic camelina overexpressing *PpJAZ1*

To investigate the effect of *PpJAZ1* overexpression on flower petal opening in camelina, the *PpJAZ1* gene [70] was stably transformed into camelina under the control of the full-length of CaMV *35S* promoter using the floral dip method [22, 76] (Supplementary Data Figure S1). A total of 83 independent T_1_ overexpression lines were obtained. Seventeen out of the 83 overexpression lines exhibited a 3:1 segregation ratio on hygromycin-containing media, indicating a single T-DNA insertion in these lines. These 17 single-copied overexpression lines were advanced to T_3_ or T_4_ generations homozygous for the transgene.

### Phenotypic analysis of the stable transgenic camelina lines overexpressing *PpJAZ1* at the vegetative and flowering stages under greenhouse conditions

**Figure 2:**
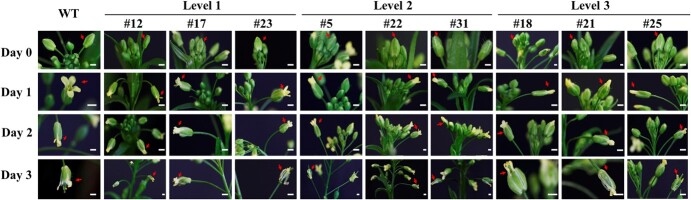
Representative images of the three levels of cleistogamy in the single-copied homozygous transgenic camelina lines overexpressing the *PpJAZ1* gene from peach. Day 0, flower bud stage (prior to flowering). Day 1, anthesis stage. Day 2, post-anthesis stage. Day 3, fruit formation stage. Red arrows, different flower developmental stages of the same flower of each line. Bar = 1 mm.

Phenotypic analysis was conducted in the 17 single-copied homozygous overexpression lines, and phenotypic difference was not observed between the transgenic and non-transgenic camelina plants before flowering. When plants began to flower, the floral developmental morphology analysis observed apparent difference in the degrees of flower petal opening between the transgenic lines and the non-transgenic camelina plants. As shown in Figure 2, we observed that camelina flowers have four developmental stages, i.e. the flower bud (Day 0), anthesis (Day 1), post-anthesis (Day 2), and fruit formation (Day 3) stages. At the flower bud stage (Day 0; prior to flowering), all the overexpression lines showed the same phenotype in their flower buds as the non-transgenic plants with tiny petal tips being barely observable (Figure 2). Hand emasculation of 20 non-transgenic plants at Day 0 showed that all the bagged emasculated flowers became abortive, indicating that self-pollination did not occur in camelina at Day 0 (Supplementary Data Figure S2).

At the anthesis stage (Day 1) when flowers are fully open, various degrees of flower petal opening were observed in these overexpression lines, which were grouped into three levels of cleistogamy (Figure 2). When compared to the non-transgenic flowers, Levels 1, 2, and 3 cleistogamy showed half opening, straight petals, and closed petals, respectively. At the post-anthesis stage (Day 2), both the transgenic and non-transgenic flowers started to wither, indicating a one-day-long life of camelina flowers. Losing water and vigor made the petals distorted and open widely. At the fruit formation stage (Day 3), flower senescence continued and petals started to fall off as the fertilized ovary started to enlarge in the overexpression lines and the non-transgenic camelina (Figure 2). As a result, the observed phenotypic difference between the overexpression lines and the non-transgenic camelina came from the degrees of cleistogamy in the overexpression lines at Day 1 only. Thus, three independent single-copied homozygous overexpression lines were randomly chosen for each of the three levels of cleistogamy for further analysis. These included lines #12, 17, and 23 for Level 1 cleistogamy, lines #5, 22, and 31 for Level 2 cleistogamy, and lines #18, 21, and 25 for Level 3 cleistogamy (Figure 2).

### Molecular analysis of the stable transgenic camelina lines overexpressing *PpJAZ1*

PCR amplification was used to confirm the presence of the *PpJAZ1* transgene in the nine overexpression lines by using transgene-specific primers. As shown in Supplementary Data Figure S3, all the nine overexpression lines were PCR positive, indicating the transgene was successfully integrated into the genome of each overexpression line.

**Figure 3:**
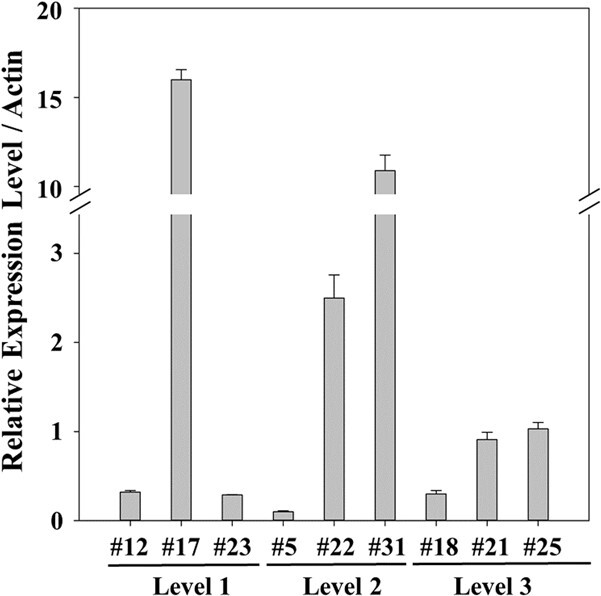
The relative expression levels of the transgene *PpJAZ1* in the leaves of transgenic camelina lines overexpressing *PpJAZ1* measured by qPCR. The camelina *Actin* gene was used as the reference gene. qPCR was conducted as described in Zhao *et al.* [77] and data analysis was conducted using the 2^−ΔΔCt^ method. The mean values of three independent replicates ± standard errors (vertical bars) are displayed.

Real-time RT-PCR (qPCR) was used to measure the relative expression levels of the *PpJAZ1* transgene in each of the nine overexpression lines using our newly developed method [77] and the *Actin* gene (accession #: XM 010467690.2) as the internal control gene [78]. As shown in Figure 3, qPCR analysis of the nine overexpression lines showed up to 20-fold overexpression in leaves but various levels of relative expression among different lines. The relative expression levels of the *PpJAZ1* transgene in different lines were not tightly correlated with the levels of cleistogamy in these lines, possibly because *JAZ1* is a master gene regulating the expression of multiple genes, which has been observed for the *Myb4* gene in transgenic switchgrass [79].

### The effect of *PpJAZ1* overexpression on seed yield under greenhouse conditions

**Figure 4:**
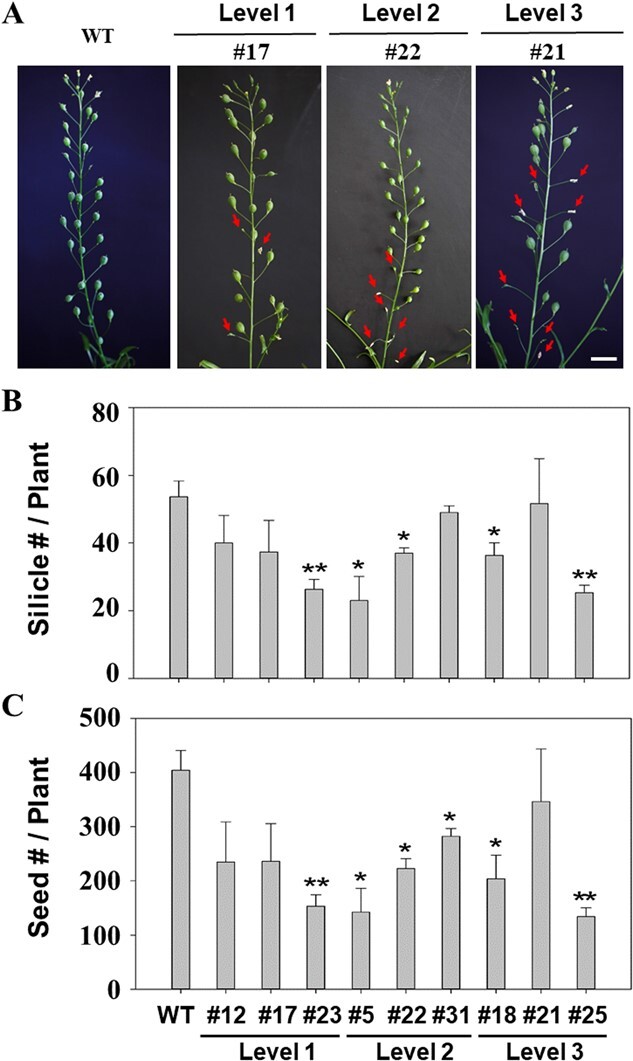
The effect of the overexpressed *PpJAZ1* gene on the silicle development on the main branches (A), silicle number per plant (B), and seed number per plant (C) of the transgenic camelina lines at Days 1 ~ 3. WT, non-transgenic. Red arrows, the aborted silicles on the main branches. Bar = 1 cm.

When compared with the non-transgenic plants, the transgenic lines exhibited a visible difference in fruit abortion under greenhouse conditions (Figure 4A). Fruit abortion was only observed on the main branches of the transgenic lines and was apparently correlated with the levels of cleistogamy. For instance, Level 1 cleistogamy had 2–3 aborted fruit silicles (pods) per plant, Level 2 cleistogamy showed 5–6 aborted silicles per plant, while Level 3 cleistogamy exhibited 8–9 aborted silicles per plant (Figure 4A).

To further evaluate the effects of the overexpressed *PpJAZ1* on seed yield, we counted the silicle number per plant and seed number per plant. When compared to the non-transgenic plants, up to 20 ~ 50% decrease in silicle number per plant and seed number per plant was identified in some transgenic lines (Figures 4B; 4C). Among the nine overexpression lines, we found that only one of the three lines of Level 1 cleistogamy (line #23) exhibited a significant decrease in silicle number per plant and seed number per plant when compared to the non-transgenic plants. However, at least two of the three lines of Levels 2 and 3 cleistogamy showed a significantly less silicle number per plant and seed number per plant than the non-transgenic plants. Interestingly, line #21 from Level 3 cleistogamy did not exhibit a significant difference in silicle number per plant and seed number per plant from the non-transgenic plants.

We also measured one hundred seed weight and found that there was no significant difference in the one hundred seed weight between the non-transgenic camelina and the overexpression lines (Supplementary Data Figure S4).

### The effect of *PpJAZ1* overexpression on pollen viability under greenhouse conditions

To investigate whether the overexpressed *PpJAZ1* affected pollen viability, we examined the pollen germination rates *in vitro* as a proxy to estimate pollen viability. Since hand emasculation revealed that self-pollination does not occur in camelina at Day 0 Figure 3 (Figure S2), all of the pollen samples were collected daily from each of the nine overexpression lines and the non-transgenic plants at Days 1 ~ 3. There was no apparent difference in the development of stamens and anther including pollen shape and size between the transgenic and non-transgenic plants when viewed under the light microscope (data not shown).

**Figure 5:**
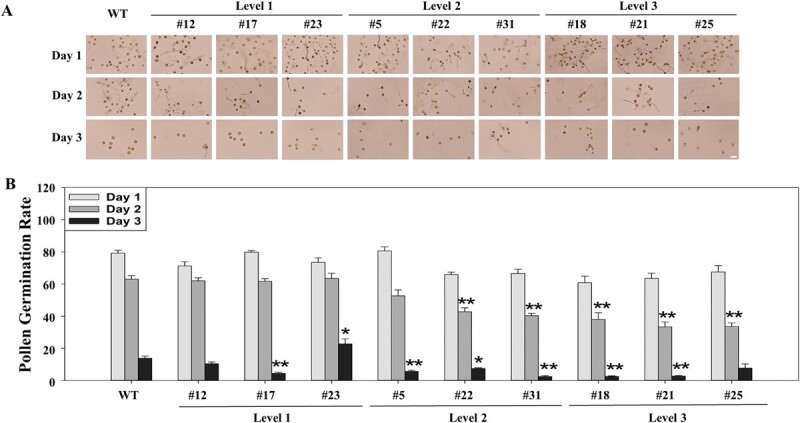
The effect of the overexpressed *PpJAZ1* gene on pollen germination rates of the transgenic camelina lines at Days 1 ~ 3. (A) Representative images of pollen germination. (B) Pollen germination rate. WT, non-transgenic. Bar = 50 μm.

When cultured on the optimized pollen germination medium for 24 hours, the pollen germination rates were 60 ~ 80% for pollen collected at Day 1, 40 ~ 60% for pollen collected at Day 2, and 2 ~ 20% for pollen collected at Day 3 for all the overexpression lines and the non-transgenic plants (Figure 5). For pollen collected at Day 1, pollen germination rates were insignificantly different between all the overexpression lines (60% ~ 80%) and the non-transgenic plants (70% ~ 80%). For pollen collected at Days 2 and 3, however, five and seven out of the nine overexpression lines exhibited significantly lower pollen germination rates than the non-transgenic plants, respectively. For example, the pollen germination rate at Day 2 was 60% for pollen collected in the non-transgenic plants, while that was 60%, 45%, and 40% for pollen collected from each of the Levels 1, 2, and 3 cleistogamous lines, respectively. Similarly, the pollen germination rate at Day 3 was 15% for pollen collected in the non-transgenic plants, while that was 17%, 10%, and 5% for pollen collected from Levels 1, 2, and 3 cleistogamous lines, respectively. These results indicate that the overexpressed *PpJAZ1* significantly affected pollen viability at Days 2 and 3 when flower finished anthesis and petals started to fall off. It is worthwhile to point out that the pollen germination rate dropped to 33% and 3% for pollen collected in line #21 at Days 2 and 3 (Figure 5B). Since line #21 also showed insignificant difference in silicle number per plant and seed number per plant when compared to the non-transgenic plants (Figures 4B;4C), it was chosen for field trial studies.

### The effect of *PpJAZ1* overexpression on PMGF during field trials

**Figure 6:**
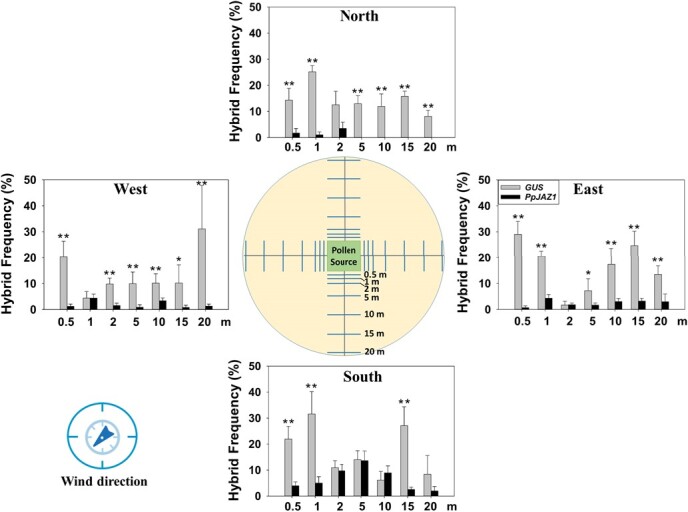
The effect of the engineered cleistogamy in the best transgenic camelina line on PMGF under the field conditions. One field site per transgene was used for the transgenic camelina line overexpressing the *PpJAZ1* or *GUSPlus* reporter gene. In each field site, the best transgenic camelina line was planted for each transgene in the center of 5 × 5 m as the pollen source, while the non-transgenic camelina plants were planted as the pollen recipients in the four directions with distances of 0.5, 1, 2, 5, 10, 15, and 20 m for each direction. Seeds were harvested from all of the non-transgenic camelina plants at each distance in each direction on each field site, and used for germination on solid media containing the proper antibiotics for selection, followed by PCR confirmation of the presence of the transgene in the antibiotics-resistant seedlings.

To investigate the effect of the overexpressed *PpJAZ1* on PMGF under open-field conditions, two field trials were conducted with *PpJAZ1* overexpression line #21 on one field site and a single-copied homozygous transgenic camelina line overexpressing the *GUSPlus* reporter gene on a second field site (Supplementary Data Figure S5). The field trial of the *GUSPlus* overexpression line was used as the negative control since the overexpressed *GUSPlus* does not change flower petal opening and gentamycin was used for transgenic plant selection. Using a modified Nelder wheel design [80], the *PpJAZ1* or *GUSPlus* overexpression line was planted in the center of each field site and used as the pollen donor. The non-transgenic camelina plants were planted along each of the four rays (east, south, west, and north) of each field site at set distances (i.e. 0.2, 1, 2, 5, 10, 15, and 20 m from the edges of the pollen donors [40]) and used as the pollen recipients (Figure 6; Supplementary Data Fig. S4). Weekly monitoring and analysis of field plots did not show apparent difference in agricultural traits including seed germination rate, plant height, leaf shape, flower opening pattern, flowering date, petal shape, petal number, silicle maturity date, and disease rating between transgenic and the non-transgenic plants in the field. All the plants started to flower at the end of March and seeds matured at the end of May.

Seeds were collected from all the non-transgenic plants (pollen recipients) at each distance in each ray of each field site. Three replicates of about 170 seeds per replicate were randomly chosen from each distance in each ray for each field site to germinate on MS solid media containing hygromycin (for *PpJAZ1*) or gentamycin (for *GUSPlus*). Hygromycin- or gentamycin-resistant seedlings should result from the pollination of the transgenic pollen on the non-transgenic stigmas. PCR amplification was used to confirm the presence of each transgene in the antibiotics-resistant seedlings. Thus, we used the hybrid ratio, which was calculated by dividing the number of antibiotics-resistant and PCR-positive seedlings by the total number of seedlings germinated for each distance in each ray for each field site, as a proxy to estimate PMGF from the transgenic plants to the non-transgenic plants within each field site.

For the *PpJAZ1* overexpression line, the hybrid ratios in the south were apparently higher than that in the east and west, while each of the latter two was apparently higher than that in the north (Figure 6). For example, the highest hybrid ratio in the south was detected at the distance of 5 m (13.6%), followed by the distances at 2 (10.9%), 10 (8.9%), 1 (5.0%), 0.5 (4.0%), 15 (2.6%) and 20 m (0.9%). However, the highest hybrid ratio in the north was identified at the distance of 2 m (3.5%), followed by the distances at 0.5 (1.7%) and 1 (1.0%), and that were 0% at the distances at 5 ~ 20 m. The hybrid ratios in the east were comparable to that in the west, ranging from 0.7% to 4.4% for all the distances. These data are consistent with the prevailing southwest wind direction of the growing season in the field (Figure 6).

When compared to the *GUSPlus* control line, the hybrid ratio for the *PpJAZ1* overexpression line was dramatically lower (Figure 6). For the south, the hybrid ratios for the distances at 0.5, 1, and 15 m for the *PpJAZ1* overexpression line was 4.0%, 5.0%, and 2.6%, respectively, which was significantly less than their counterparts for the *GUSPlus* control line (i.e. 21.9%, 31.6%, and 27.1%, respectively). Similarly, a significant difference in the hybrid ratios was identified for the distances at 0.5, 1, 5, 10, 15, and 20 m for the east, for the distances at 0.5, 2, 5, 10, 15, and 20 m for the west, and for the distances at 0.5, 1, 5, 10, 15, and 20 m for the north.

As a result, the overexpressed *PpJAZ1* dramatically restricted PMGF under the field conditions.

## Discussion

PMGF from transgenic plants to non-transgenic plants and wild relatives results in adverse effects on the environment. Effective and reliable bioconfinement methods are essential to prevent PMGF in the field. Here, we developed a highly effective bioconfinement approach to limit PMGF from transgenic camelina to non-transgenic camelina under the field conditions using the overexpressed *PpJAZ1* gene in transgenic camelina. Substantial PMGF has been detected in camelina in the field [39, 40], making the development of effective bioconfinement approach a prerequisite for deploying transgenic camelina in the field. In the present study, we detected a maximum PMGF of 31.6% for the *GUSPlus* overexpression line (normal petal opening) at the sampling distance of 1 m (Figure 6), which was much higher than the detected maximum PMGF of 0.78% in Walsh *et al.* [40]. Thus, our results further confirmed the high outcrossing rate between camelina plants in the field and indicated that the outcrossing rate in camelina should be high for commercial field production. The difference in the PMGF values between both studies may indicate that many factors affect PMGF in camelina in the field, e.g. the population size of the donor plants and the receptor plants, weather conditions (temperature, humidity, wind direction, wild speed), and flowering time. Even though our data were from one-year field trials, we expect that our conclusion of the effect of the *PpJAZ1*-mediated cleistogamy on PMGF will not change if multiple years of field trials would be conducted.

More importantly, we found that the engineered cleistogamy in camelina dramatically inhibited PMGF under the field conditions since the hybrid ratios for the *PpJAZ1* overexpression line ranged from 0.0% to 13.6% under the field conditions, signifying the efficacy of an efficient bioconfinement approach for transgenic camelina production. Our approach might be better than previously reported bioconfinement methods such as male sterility, plastid transformation, and transgene excision since our engineered cleistogamy in the best-performing line does not interfere with pollen germination rate during anthesis and the silicle number and seed number per plant, but dramatically decreases pollen germination rates after anthesis (Figures 5 and 6). The significantly decreased pollen germination rates after anthesis are important since petals start to wither and fall off at Days 2 and 3, which may help with pollen release. Thus, the present study is the first report of using cleistogamy for bioconfinement in a dicot species under the field conditions, which should be potentially used for bioconfinement in other self-pollinated dicot species.

Ectopic expression of *PpJAZ1* induced cleistogamy in camelina in the present study and tobacco in Sherif *et al.* [70] indicate a conserved function of *PpJAZ1* in flower opening. The present study is the first to show that *PpJAZ1* overexpression significantly affected pollen germination in Days 2 and 3 but not Day 1. This is consistent with a previous report that the ectopic expression of *AtJAZ1* lacking the Jas domain caused sterile pollen in Arabidopsis since the Jas domain is important for JA signaling transduction and protein interaction [75]. The JA biosynthesis gene *ACX1* and *DAD1* also affect the flower development and petal expansion in Chinese cabbage and Arabidopsis [71, 73, 75, 81]. The function of *PpJAZ1*-mediated cleistogamy in camelina will shed light on the potential application of the *JAZ1* gene in the PMGF in plants.

We did observe several aborted silicles on the main branches of some transgenic camelina lines due to the overexpression of *PpJAZ1*, leading to the significantly decreased silicle number per plant and seed number per plant in some transgenic lines (Figure 4). The underlying mechanism of the *PpJAZ1* overexpression-induced silicle abortion remains unknown even though it could be overcome by using flower-specific (e.g. the tomato *MADS-box 6* (*TM6*) promoter [82]) or inducible promoters [83–85]. It is also worth noting that the *PpJAZ1* gene could be used together with other bioconfinement strategies, e.g. male sterility, transgenic mitigation, or maternal inheritance [48, 49, 86, 87] for gene/trait stacking to further limit PMGF from transgenic plants.

## Experimental procedures

### Plant materials and growth condition

Camelina var. Calena was used for genetic engineering in the present study. The non-transgenic and transgenic camelina plants were grown in growth chambers at 23°C with a 16/8 hour light/dark photoperiod (500 μE m^−2^ s^−1^). The plants were watered as needed and fertilized weekly with soluble fertilizer Peters® Professional (Everris; Dublin, OH, USA). Monterey Garden Insect Spray (Lawn and Garden Products; Fresno, CA, USA) and Rose & Flower Insect Killer (SBM Life Science Corp; Cary, NC, USA) were used to control thrips and aphids every two weeks or as needed according to the manufacturer’s instructions.

### Vector construct

The *GUSPlus* reporter gene was PCR amplified from the pCAMBIA1305.2 and cloned into the pZP35S:RFP vector (containing a gentamycin resistance gene for transgenic plant selection) [88] to replace the RFP reporter gene with the help of *Bam*HI and *Hind*III. The cDNA sequence of the cleistogamy-inducing *PpJAZ1* gene (Accession #: EMJ03624) from peach was PCR amplified from the binary vector pGrII-35S-PpJAZ1-eGFP [70], fused in-frame with a red fluorescent protein *pporRFP* reporter gene [88–90], and cloned into the pCR8 vector (Thermo Fisher; Waltham, MA, USA). Following Sanger sequencing, the *PpJAZ1-pporRFP* was cloned into the binary vector pMDC32 (containing a hygromycin resistance gene for transgenic plant selection) under the control of the full-length CaMV *35S* promoter using the LR reaction (Thermo Fisher; Waltham, MA, USA). The resulting destination plasmid was confirmed using Sanger sequencing, and named as the pMDC35S JAZ1-RFP (Supplementary Data Figure S1). The primers used in PCR amplification were listed in Supplementary Data Table S1.

### Plant transformation

Camelina transformation was performed using the floral dip method as described previously [22, 76] with modifications. The binary vectors pMDC35S JAZ1-RFP and pZP35S GUSPlus were transformed into *Agrobacterium tumefaciens* strain GV3850 individually. The *Agrobacterium* colonies were selected on YEP solid media containing rifampicin (50 mg/L) and kanamycin (50 mg/L) or spectinomycin (100 mg/L) at 28°C for 2–3 days. The positive colonies were verified by PCR and Sanger sequencing and restreaked twice and then grown in 5 mL liquid YEP (5 g/L yeast extract, 10 g/L peptone, 5 g/L NaCl; pH 6.8) containing antibiotics. Overnight culture of the 5 mL *Agrobacterium* was transferred into a 1 L flask containing 500 ml of the same YEP medium and incubated for 16 h at 28°C. The *Agrobacterium* suspension was centrifuged at 4,000 rpm for 10 min and re-suspended in infiltration medium (pH 5.7) consisting of half strength Murashige and Skoog Salt Mixture powder (MS) basal salts, 50 g/L sucrose, 2 mg/L benzylaminopurine (BAP) in dimethyl sulfoxide (DMSO), and 0.05% (v/v) Silwet L77 (Lehle Seeds; Round Rock, TX, USA) prior to plant transformation. Flowering camelina plants were placed in a 65-L capacity vacuum desiccator (Bel-Art-SP Scienceware; Wayne, NJ, USA) and the inflorescence shoots were dipped in the *Agrobacterium* suspension culture in a 1,000-mL plastic box. The vacuum desiccator was slowly brought to a pressure of 80 psi and held for 5 minutes. Then, the plants were kept under black plastic bags in darkness for 24 h, followed by being transferred to growth chamber under normal growing conditions for seed harvest (T_1_ seeds).

### Phenotypic and molecular analysis of transgenic plants

T_1_ seeds of the transgenic camelina plants overexpressing *PpJAZ1-pporRFP* were harvested and selected on MSO solid medium with hygromycin (50 mg/L) and Timentin (400 mg/L). T_1_ seeds of the transgenic camelina plants overexpressing *GUSPlus* were harvested and selected on MSO solid medium with gentamycin (300 mg/L) and Timentin (400 mg/L). Non-transgenic camelina cv. Calena was used as the negative control. Potential transgenic T_1_ lines were selected based on the root length and shoot length when compared with non-transgenic plants, and transferred to soil for growth to flowering.

These potential transgenic lines were subjected to visual phenotypic screening for the cleistogamous phenotype at the floral stage. The seeds from the lines with cleistogamous phenotype were screened on the same MSO plates, and the 3:1 antibiotic resistance segregating (i.e. single-copied) lines were advanced into next generations in greenhouse for homozygosity. Silicle number per plant and seed number per pod were counted and recorded. One hundred seed weight were measured using a Mettler-Toledo ME54TE analytical balance (Mettler-Toledo; Mettler Toledo, Schwerzenbach, Switzerland).

The single-copied homozygous lines were subjected to PCR confirmation of the presence of the transgene using the transgene-specific primers (Supplementary Data Table S1). The genomic DNA were extracted from 100 mg young leaf of each line using the CTAB method [91]. The DNA concentration was measured by Nanodrop ND-1000 spectrophotometer (NanoDrop Technologies; Wilmington, DE, USA). PCR reactions were conducted with an initial denature at 94°C for 3 min, followed by 40 cycles of 94°C 30 s, 58°C 30 s, 72°C 60 s and an extension of 72°C 10 min. PCR amplicons were analyzed by gel electrophoresis.

### RNA isolation and cDNA synthesis

The single-copied homozygous lines were also subjected to qPCR analysis of relative transgene expression. Total RNA was extracted from 100 mg young leaf tissue of each line using the TRIzol reagent (Molecular Research Center; Cincinnati, OH, USA) according to manufacturer’s instructions. Three biological replicates were used for each line. DNase I (New England Biolabs; Ipswich, MA, USA) was used to remove the contaminated genomic DNA, followed by RNA purification using the GeneJET RNA Cleanup and Concentration Micro Kit (Thermo Fisher; Waltham, MA, USA). RNA concentration and purity was measured by Nanodrop ND-1000 spectrophotometer, followed by gel electrophoresis.

cDNA synthesis was performed from 1 μg of total RNA using the SuperScript III First-Strand Synthesis System (Thermo Fisher; Waltham, MA, USA), 1 μl oligo(dT) primers (50 μM), 1 μl 10 mM dNTPs and DEPC-treated water in a final volume of 10 μl. The mixture was incubated at 65°C for 5 min and then on ice for 1 min. The following cDNA synthesis mixture was prepared by addition of 2 μl 10 × RT buffer, 4 μl MgCl_2_, 2 μl DTT, 1 μl RNase OUT™ and 1 μl SuperScript™ III RT. The mixture was incubated at 50°C for 50 min, and 85°C for 5 min. The synthesized cDNA was stored in −20°C.

### qPCR

qPCR was performed in a CFX96 Touch Real-Time PCR detection system (Bio-Rad Laboratories; Hercules, CA, USA) using the FastStart Universal SYBR Green Master (Roche Diagnostics Corporation; Indianapolis, IN, USA) as described in Zhao *et al*. [77] and Duduit *et al.* [92]. The optimal annealing temperature, primer concentration, and appropriate cDNA concentrations were determined to get the lowest Cycle threshold (Ct) value as the optimal conditions prior to the qPCR. Temperature gradient PCR was used to test the optimal annealing temperature under the diluted cDNA concentration (1:10 dilution) and 350 nM primers. The optimal annealing temperature was set at the lowest Ct value in the gradient PCR and used for the optimal primer concentration screening. The standard cDNA concentration curve with a logarithmic scale was determined by serial dilutions of the cDNA (1:10, 1:20, 1:40, 1:80, 1:160 dilution). The camelina *Actin* gene (accession *Csa19g026200*) was used as the reference gene. Three biological replicates were used with three technical replicates to minimize the systematic error.

### Floral emasculation experiment

In order to determine whether selfing occurs in unopened camelina flowers in Day 0, the filaments of the non-transgenic camelina flowers were completely removed (emasculated) and bagged in Day 0. Fruit silicles were counted and photographed in Day 4.

### *In vitro* pollen germination experiment

Pollen was collected from fully opened flowers of non-transgenic Calena and single-copied homozygous transgenic lines in Day 1, 2 or 3, and germinated on pollen germination medium containing 15% sucrose, 5 ppm boric acid, and 0.5% agar at room temperature in the dark for 24 hours. Pollen germination rate was counted under the light microscope (Nikon; Minato City, Tokyo, Japan).

### Field trials of PMGF

To measure PMGF from transgenic to non-transgenic camelina plants, the field trial experiments were carried out at Sandhills Research Station, Jackson Springs, NC (35.18782°N 79.68°W) from March 20, 2020 to July 10, 2020. Two field sites which were ≥ 100 m away from each other were used. Herbicides and fertilizer were applied to each field prior the field trial. A modified field design was used in the field experiment [78]. The best single-copied homozygous plants overexpressing *PpJAZ1* or *GUSPlus* were used as the pollen donors and planted in rows by hand in the source square (5 × 5 m) with 50-cm row spacing [39]. The non-transgenic pollen recipient plants were planted at distances of 0.5, 1, 2, 5, 10, 15, and 20 m in the four directions from the pollen donors [40]. Both transgenic and non-transgenic seeds were planted at a depth of ~1 cm. All seeds were collected and harvested from the pollen recipient plants at each distance in each direction of each field site. The seeds collected from the recipient plants were germinated on germination plate with selected antibiotics, and seed germination rate was recorded. PCR amplification was used to confirm the presence of each transgene in the antibiotics-resistant seedlings.

### Statistical analysis

Statistical analysis was performed using the software SAS (*p* ≤ 0.05; SAS 9.2 for Windows; SAS Institute, Cary, NC).

## Supplementary Material

Web_Material_uhac280

## Data Availability

All the data supporting the findings of the present study are available within the paper and its supplementary data.
