# Supplementary material for: Engineered Cleistogamy in Camelina sativa for bioconfinement
Source: Hortic Res. 2022 Dec 22;10(2):uhac280. doi: 10.1093/hr/uhac280 (PMC9926159; doi:10.1093/hr/uhac280)

Supplementary data

Table S1. Primer sequences used in the present study

**Primer**	**Sequence (5’ > 3’)**	**Purpose**
PpJAZ1-F	ATGTCGGAGTTCATCGGAGACT	Cloning
PpJAZ1-RFP-R1	CCACTTTGCTTTGAAAGAGCCATTTGGGTT GGTTGAGCAGCC	Cloning
PpJAZ1-RFP-F1	TGGCTGCTCAACCAACCCAAATGGCTCTT TCAAAGCAAAGTGG	Cloning
RFP-R	TTAGGCTACCGGTAAGTTAGAAAGATGT	Cloning
GUSPlus-BamH I- F	ATGGATCCATGGCTACTACTAAGCAT	Cloning
GUSPlus-Hind III R	CTAAGCTTGATCTAGTAACATAGATG	Cloning
PpJAZ1-qRT-F	CTTCACCGATTCCTGGAGAAG	qPCR
PpJAZ1-qRT-R	CAGCCAAGCCTAACCATGAC	qPCR
CsActin2-qRT-F1	GTGGATATCAGGAAGGATCTA	qPCR
CsActin2-qRT-R1	GATTTCTTTGCTCATACGGTCT	qPCR
CsActin2-qRT-F2	CTATGTTCTCTGGTATCGCA	qPCR
CsActin2-qRT-R2	AGACACTGTACTTCCTTTCC	qPCR

Figure S1. Plasmid maps of the *35S:PpJAZ1* (A) and *35S:GUSPlus* (B) vectors used in the present study. HygR, hygromycin resistance gene. GentR, gentamycin resistance gene.

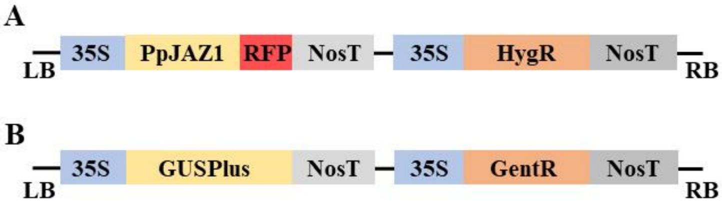

Figure S2. The effect of emasculation before anthesis (i.e., Day 0) on the silicle development in non-transgenic camelina plants. Bar = 2 mm.

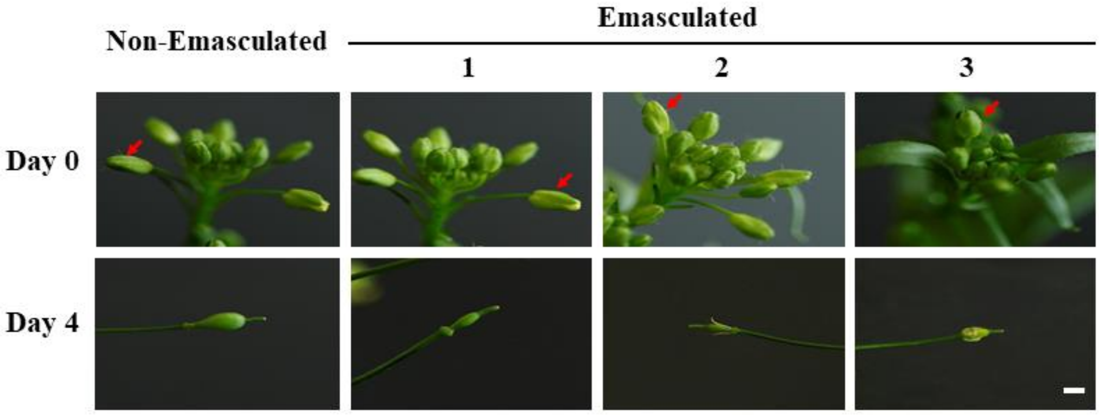

Figure S3. PCR confirmation of the presence of the transgene *PpJAZ1* in the transgenic camelina lines. WT, non-transgenic.

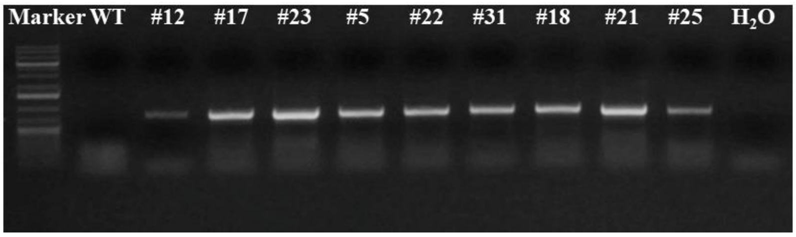

Figure S4. The effect of the overexpressed *PpJAZ1* gene on one hundred seed weight of the transgenic camelina lines. WT, wild-type (non-transgenic).

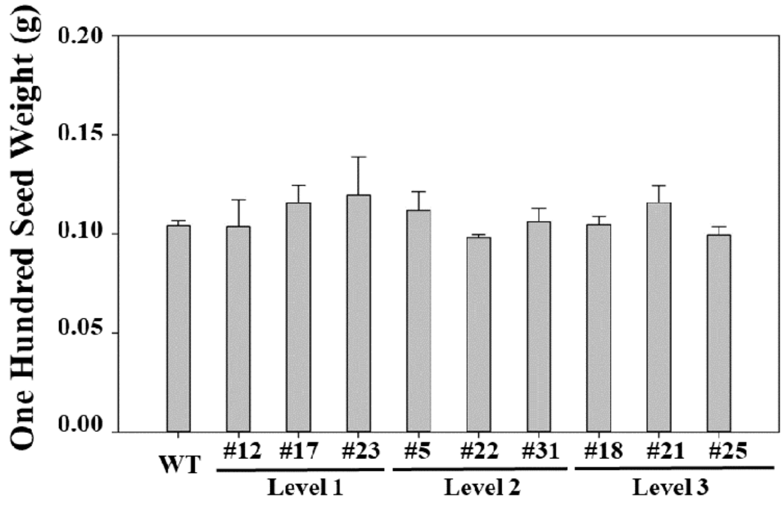

Figure S5. Overview of the plantation of one field site. (A) Six-week old camelina seedlings. (B) Eight-week old camelina seedlings. (C) Flowering camelina plants. (D) Mature camelina plants.

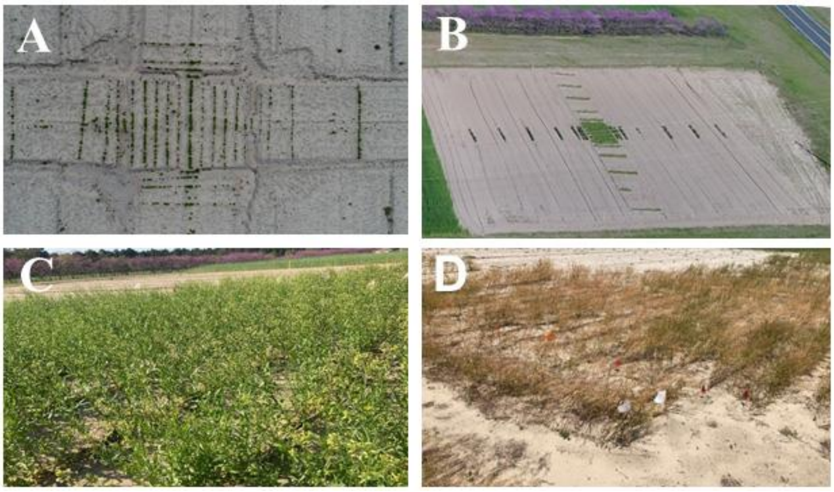

Supplement: Web_Material_uhac280 [file web_material_uhac280.pdf]
